# Effects of hypo-*O*-GlcNAcylation on *Drosophila* development

**DOI:** 10.1074/jbc.RA118.002580

**Published:** 2018-03-27

**Authors:** Daniel Mariappa, Andrew T. Ferenbach, Daan M. F. van Aalten

**Affiliations:** From the Division of Gene Regulation and Expression, School of Life Sciences, University of Dundee, Dundee DD1 5EH, Scotland, United Kingdom

**Keywords:** O-linked N-acetylglucosamine (O-GlcNAc), O-linked N-acetylglucosamine (O-GlcNAc) transferase (OGT), Drosophila genetics, CRISPR/Cas, polycomb, post-translational modification (PTM), Drosophila development, Host cell factor, hypomorphic sxc mutant, Mediator complex

## Abstract

Post-translational modification of serine/threonine residues in nucleocytoplasmic proteins with GlcNAc (*O*-GlcNAcylation) is an essential regulatory mechanism in many cellular processes. In *Drosophila*, null mutants of the Polycomb gene *O*-GlcNAc transferase (*OGT*; also known as super sex combs (*sxc*)) display homeotic phenotypes. To dissect the requirement for *O*-GlcNAc signaling in *Drosophila* development, we used CRISPR/Cas9 gene editing to generate rationally designed *sxc* catalytically hypomorphic or null point mutants. Of the fertile males derived from embryos injected with the CRISPR/Cas9 reagents, 25% produced progeny carrying precise point mutations with no detectable off-target effects. One of these mutants, the catalytically inactive *sxc^K872M^*, was recessive lethal, whereas a second mutant, the hypomorphic *sxc^H537A^*, was homozygous viable. We observed that reduced total protein *O*-GlcNAcylation in the *sxc^H537A^* mutant is associated with a wing vein phenotype and temperature-dependent lethality. Genetic interaction between *sxc^H537A^* and a null allele of *Drosophila* host cell factor (*dHcf*), encoding an extensively *O*-GlcNAcylated transcriptional coactivator, resulted in abnormal scutellar bristle numbers. A similar phenotype was also observed in *sxc^H537A^* flies lacking a copy of skuld (*skd*), a Mediator complex gene known to affect scutellar bristle formation. Interestingly, this phenotype was independent of OGT Polycomb function or dHcf downstream targets. In conclusion, the generation of the endogenous OGT hypomorphic mutant *sxc^H537A^* enabled us to identify pleiotropic effects of globally reduced protein *O*-GlcNAc during *Drosophila* development. The mutants generated and phenotypes observed in this study provide a platform for discovery of OGT substrates that are critical for *Drosophila* development.

## Introduction

Nucleocytoplasmic post-translational modification of protein serine/threonine residues with GlcNAc, otherwise known as *O*-GlcNAcylation, is a key regulator of several cellular signaling events (1). *O*-GlcNAc transfer is mediated by *O*-GlcNAc transferase (OGT),^3^ whereas *O*-GlcNAcase (OGA) removes the modification from proteins. The OGT donor substrate UDP-GlcNAc is one of the critical regulators of *O*-GlcNAcylation and is a product of the hexosamine biosynthetic pathway (2). Change in flux through the hexosamine biosynthetic pathway downstream of glucose availability leads to altered UDP-GlcNAc levels and consequently impinges upon levels of nucleocytoplasmic protein *O*-GlcNAcylation (3). Thus, *O*-GlcNAc signaling is an important transducer of cellular glucose levels, modulating the function of the *O*-GlcNAcylated substrates by multiple mechanisms, including changes in enzyme activity (4), protein stability (5, 6), oligomerization (7), and solubility (8). Protein *O*-GlcNAcylation has also been demonstrated to occur co-translationally and was shown to increase the stability of nascent protein chains (9). Modulation of protein function by *O-*GlcNAcylation ultimately leads to altered transcriptional profiles (10, 11). Increasing evidence associates deregulation of *O*-GlcNAc signaling with disease states such as cancer, diabetes, and neurodegeneration (12). Point mutations in OGT that segregate with X-linked intellectual disability have recently been described (13, 14).

Loss or knockdown of *OGT* in metazoa leads to lethality at various stages of development (15–18). Mouse embryonic stem cells are not viable in the absence of *ogt*, and tissue-specific *ogt* knockout leads to a range of phenotypes in nervous and immune systems (15, 19, 20). Reduction in OGT levels in *Xenopus* and zebrafish leads to severe growth defects (17, 18). In *Drosophila*, *OGT* (also known as *sxc* (super sex combs), henceforth referred to only as *sxc*) mutants die as pharate adults (21). *sxc* is a Polycomb group (*PcG*) gene that contributes to control of *HOX* gene expression and specification of segmental identity (16). The *Drosophila* embryonic *O*-GlcNAcome is dynamic, with increased numbers of proteins becoming *O*-GlcNAc-modified with developmental time (22). Polyhomeotic (Ph), a core component of the PRC1, has been identified as a key *O*-GlcNAc substrate (8). Reduced *O*-GlcNAcylation of a Ser/Thr-rich stretch in Ph leads to its aggregation and is associated with misexpression of downstream *HOX* genes (8). Interestingly, lethality of *sxc* mutants can be rescued by transgenic overexpression of catalytically defective *Drosophila* OGT (*Dm*OGT) point mutants (23). When one of the catalytically compromised *Dm*OGT mutants, *Dm*OGT^H537A^, was used to rescue pupal lethality of *sxc* nulls, the efficiency of the rescue was about 80% relative to the rescue with *Dm*OGT^WT^. The *in vitro* catalytic activity of *Dm*OGT^H537A^ is about 6% of that of *Dm*OGT^WT^ (23). Another point mutant, *Dm*OGT^K872M^, in which the catalytic lysine residue is mutated, lacks any detectable activity *in vitro* and does not rescue pupal lethality of *sxc* mutants. These observations imply that a minimal level of protein *O*-GlcNAcylation is sufficient to support a complete life cycle in *Drosophila.* In addition, it also implies that the functionality of the most critical *O*-GlcNAc substrates in addition to Ph is still retained to a large extent in *sxc* null flies rescued by the *Dm*OGT^H537A^ mutant.

The recent emergence of CRISPR/Cas9 gene-editing technology allows the generation of flies with precise point mutations in *sxc* to begin to link phenotypes to mechanisms. Bacteria utilize CRISPR/Cas9 as a defense system against viral pathogens (24). Harnessing the endonuclease activity of Cas9 targeted to a specific genomic target by providing a single guide RNA, dsDNA breaks (DSBs) can be introduced. Repair of these DSBs by homologous recombination can be exploited to create precise point mutants. Since the first report exploiting the CRISPR/Cas9 technique to engineer targeted DSB mutants, this gene-editing strategy has been used to generate null mutants in numerous organisms (25, 26). Generation of animals with precise point mutations has been achieved in zebrafish (27) and mice (28). In *Drosophila*, CRISPR/Cas9 technology has been used to produce protein nulls (29), to create defined deletions (30), to tag proteins (31), to insert FRT/attP sites in endogenous loci (31), to activate transcription *in vivo* (32), to decipher functional implications of miRNA-miRNA response element interaction (33), and also to create a mutagenic chain reaction aimed at generating autocatalytic mutations to produce homozygous loss-of-function mutations (34). More recently, point mutants have also been generated by several groups (35–37).

Human host cell factor 1 (Hcf1) has been reported previously as an *O*-GlcNAc protein (38). A transcriptional regulator, Hcf1 is required as a host cell factor for human herpes simplex virus infection (39). Hcf1 is a large protein that is proteolytically processed by OGT into *N*-terminal Hcf1_N_ and C-terminal Hcf1_C_ products that regulate different phases of the cell cycle (40). Apart from *O*-GlcNAcylating Hcf1, mammalian OGT is also essential for this proteolytic processing of Hcf (41). Intriguingly, whereas *Drosophila* Hcf (dHcf) is also extensively *O*-GlcNAcylated (22), its proteolytic processing is instead performed by a separate protease, Taspase I (8, 22, 42). *O*-GlcNAcylation of Hcf has been proposed to prevent its aggregation (8). dHCf is a multifunctional protein, underlined by virtue of genetic interaction of a null allele, *dHcf^HR1^*, with components of the *PcG*, Trithorax (*TrxG*), and Enhancer of Trithorax and Polycomb (*ETP*) group (43). Because dHcf is not a proteolytic substrate of OGT in *Drosophila*, this is an attractive system to dissect the role of dHcf *O*-GlcNAcylation. Flies null for *dHcf* display pleiotropic phenotypes that are enhanced or suppressed in various *PcG*, *TrxG*, and *ETP* mutant backgrounds (43). Several phenotypes of the *dHcf^HR1^* mutant are enhanced by an allele of an ETP gene skuld (*skd*) (43). *skd* encodes the *Drosophila* orthologue of human MED13, a component of the Mediator complex, which is a conduit connecting transcription factor signals to RNA polymerase II transcriptional machinery (44, 45).

The effect of reduced as opposed to complete loss of protein *O*-GlcNAc at the organismal level has not been previously investigated. Here, we investigated the genetic interaction between *sxc/OGT^H537A^* and *dHcf^HR1^*, a *dHcf* null allele (43). Using hypomorphic *sxc^H537A^* homozygotes, we demonstrate that *O*-GlcNAc signaling is required for wing vein formation and tolerance to increased temperature. In addition, variation in scutellar bristle numbers is enhanced in *sxc^H537A^* mutants simultaneously lacking *dHcf* or having reduced *skd* function. In summary, these results outline the requirement of *O*-GlcNAc signaling in several pathways in *Drosophila*.

## Results

### Highly efficient gene editing with CRISPR/Cas9 generates precise sxc mutants

**Figure 1.**
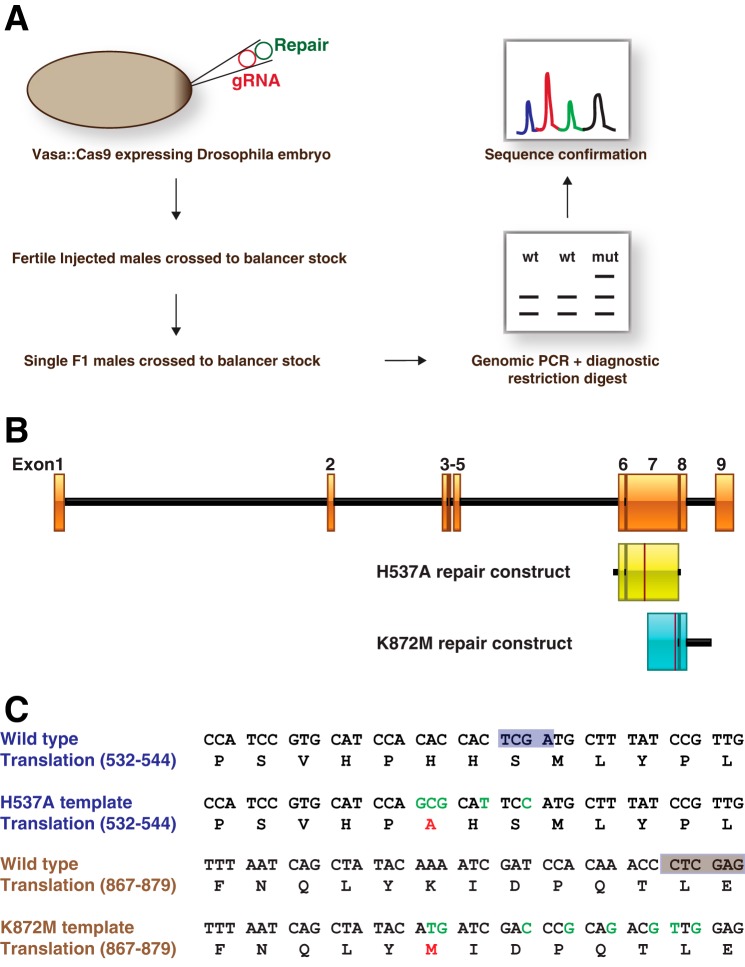
**Strategy to generate *sxc^H537A^* mutants using the CRISPR/Cas9 gene-editing technology.** *A*, experimental outline of the CRISPR/Cas9 homologous recombination scheme adopted to generate *sxc* mutant flies. gRNA and the respective homologous repair plasmids were injected into *vasa::Cas9* embryos (Bloomington stock 51323). F1 males derived from injected embryos were allowed to mate with balancer chromosome stocks, sacrificed, and genotyped using restriction fragment length polymorphism assay to determine the presence of a genetic lesion. Genomic DNA from flies that were resistant to restriction digestion was sequenced to confirm the nature of the lesion. *B*, *sxc* genomic region with exons depicted as *orange boxes* and introns as *black lines*. The extent of the genomic DNA supplied for homologous repair carrying either the H537A or the K872M mutations is shown in the *yellow* and *blue boxes*, respectively. The *red line highlighted* within each of these *boxes* marks the site of the introduced mutations in the repair constructs. *C*, genomic DNA sequence of the repair region carrying the mutation in the WT and mutant scenarios are shown. *Below* the DNA sequence is the translated protein. The changes that were made in the mutant DNA construct are *highlighted* in *green*, and the expected change in protein translation is *marked* in *red*. The TaqI and XhoI restriction sites are *marked* with *light purple* or *brown boxes*, respectively. Successful incorporation of the mutant sequence or an indel will lead to the loss of the restriction sites.

Given that *sxc* is a maternal effect gene and resides at a locus that is not amenable to producing germ line clones lacking the maternal copy using the FRT-flipase system, current approaches to eliminate the maternal copy have relied on using the UAS-GAL4 system (8, 46). To enable reliable and physiological phenotypic characterization of the requirement of the *O*-GlcNAc modification for *Drosophila* development, we embarked on producing a precise hypomorphic OGT point mutant, *sxc^H537A^* and a catalytically dead mutant, *sxc^K872M^* utilizing the CRISPR/Cas9 gene-editing technology in combination with homologous recombination (Fig. 1*A*, Table S1). Single guide RNA (sgRNA) was designed using the Zhang laboratory web tool (crispr.mit.edu).^4^ To facilitate homologous repair–based gene editing, repair constructs carrying the desired OGT hypomorphic (H537A) or catalytically dead (K872M) mutations were cloned into a pGEX6P1 plasmid (Fig. 1*B*). The homologous arms on either side of the mutations were about 1 kb long, with the repair cassette targeting exon 7 of the OGT genomic region for both of the mutations (Fig. 1*B*). In addition to the necessary mutations changing the codon to Ala in place of His at position 537 or Met in place of Lys at position 872, silent mutations were introduced in wobble positions of adjacent codons (Fig. 1*C*). This strategy was employed to decrease the chances of the repaired DNA being subjected to further Cas9 nuclease cleavage and also to enable a robust screening assay exploiting the elimination of TaqI (H537A) or XhoI (K872M) restriction enzyme sites (Fig. 1*C*).

**Figure 2.**
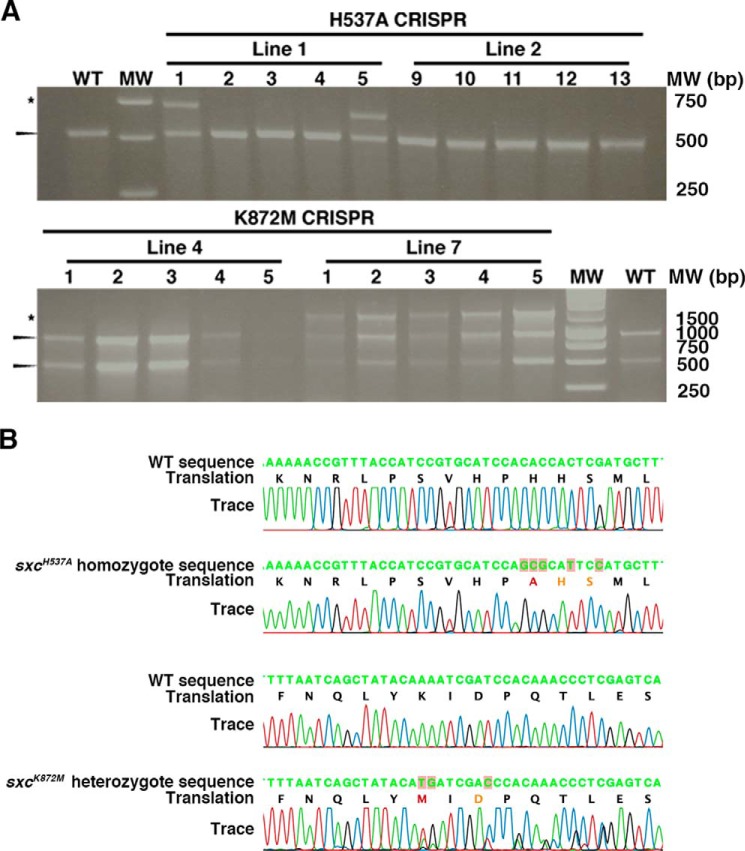
**Confirmation of *sxc^H537A^* and *sxc^K872M^* mutant lines derived by the CRISPR/Cas9 technique.** *A*, representative gels demonstrating the loss of TaqI (*above*) or XhoI (*below*) restriction sites in potential *sxc^H537A^* or *sxc^K872M^* mutants, respectively. Genomic DNA from F1 males was extracted and subjected to PCR amplification followed by restriction digest with TaqI or XhoI. Shown are restriction digests of genomic DNA from five F1 males, each derived from two injected parents. The *arrowheads* mark the digested band, whereas the *asterisk* marks the band resistant to TaqI (*above*) or XhoI (*below*). *B*, sequencing chromatograms of WT (*top*), the putative *sxc^H537A^* homozygote line 1.5 genomic DNA (*second*), WT (*third*), and the putative *sxc^K872M^* heterozygote line 7.11 (*bottom*). These data confirm the incorporation of a desired mutation that would lead to the His-537 to Ala mutation in addition to the two silent mutations that were introduced into the wobble positions in the adjacent codons. For the Lys-872 to Met mutants, the presence of multiple peaks in the chromatogram demonstrates the heterozygosity of the locus.

Both the sgRNA and the repair plasmids were injected into the *vasa::Cas9* fly line (47). Injected adult males were mated with balancer chromosome stock to eliminate the X chromosome carrying the Cas9 transgene and to balance the putative mutant chromosome. F1 males resulting from this cross were allowed to mate before sacrificing and isolating whole genomic DNA. Isolated genomic DNA was subjected to PCR followed by restriction analyses with TaqI (H537A) or XhoI (K872M). At least five individual F1 males from each of the 23 (H537A) and 8 (K872M) fertile parental lines were assessed in this manner (Table 1). A representative gel demonstrating the restriction assay from two different parental lines for each mutation is shown in Fig. 2*A*. Two lines were positive with the XhoI restriction assay while screening for the K872M mutation. Sequencing the PCR product confirmed that at least one F1 male from each of these two parental lines was positive for the precise K872M mutation. Thus, the efficiency of generating the K872M precise point mutation was 25%. Neither of the *sxc^K872M^* lines produce homozygotes or complement the well-characterized *sxc* null alleles, *sxc^1^* or *sxc^6^* (48). The *sxc^K872M^* is therefore a recessive lethal allele. Thus, the successful generation of such an allele using the CRISPR/Cas9 technique implies that loss of OGT catalysis can be tolerated during male germ cell development.

**Table 1:** **Efficiency of generating *sxc^H537A^* mutants using a CRISPR/Cas9 approach**

Mutant	Parental lines tested, PCR + restriction digestion	Precise mutations	Indels	Efficiency of precise mutation %
H537A	23	6	4	26
K872M	8	2	2	25

Screening for the H537A mutation revealed a total of six lines that were positive in the TaqI restriction assay. Sequencing showed that at least one F1 male from each of these six parental lines was positive for the precise H537A mutation, establishing the rate of generating a precise mutation at 26%. In addition, four of the six lines also carried insertions/deletions leading to *sxc* null. From the parental line 1, one of the lines that triggered the TaqI assay (line 1.1) was assessed by genomic sequencing and was found to have a 63-bp insertion resulting in a frameshift that would only code for an OGT truncation (residues 1–537). Line 1.1 did not complement either the *sxc^1^* or *sxc^6^* alleles and was found to be recessive lethal. On the other hand, sequencing of line 1.5 heterozygotes confirmed that it was a precise H537A mutation, henceforth referred to as *sxc^H537A^. sxc^H537A^* homozygotes could be derived, and their mutant status was further confirmed by sequencing (Fig. 2*B*). The codon specifying the His-537 to Ala mutation and the additional wobble mutations were also present in the homozygous *sxc^H537A^* mutants. Furthermore, upon sequencing the entire region of the ∼2-kb homologous recombination genomic boundaries, we did not observe nonspecific mutation(s) that might have been introduced during the gene-editing process. A key concern with the use of any gene-editing approach is the possibility of off-target mutagenesis. All of the potential off-targets predicted by the web tool used for gRNA selection were sequenced in the *sxc^H537A^* (Table S2) and *sxc^K872M^* (Table S3) mutants and confirmed to be WT. Thus, we have achieved highly efficient gene editing with CRISPR/Cas9 to generate *sxc* hypomorphic mutants in an otherwise endogenous background that will help interrogate the function of *O*-GlcNAc in development.

### Reduced O-GlcNAcylation is associated with wing vein phenotype and developmental lethality

**Figure 3.**
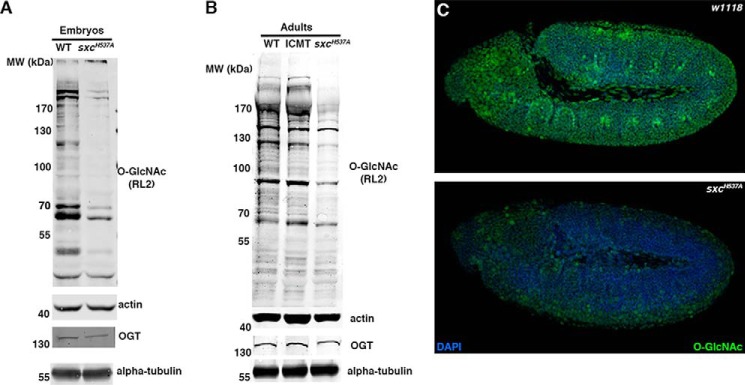
**The hypomorphic *sxc^H537A^* mutants have reduced *O*-GlcNAc levels.** *A*, *O*-GlcNAc levels are severely reduced in *sxc^H537A^* embryos. Either WT or *sxc^H537A^* homozygous embryos were collected, dechorionated, lysed, and subjected to SDS-PAGE and immunoblotted with anti-*O*-GlcNAc (RL2) or anti-OGT antibodies. The blots were normalized with either anti-actin or anti-α-tubulin antibodies, respectively. *B*, *O*-GlcNAc levels are severely reduced in *sxc^H537A^* adults. WT, balancer (*IF/CyO; MKRS/TM6* (*ICMT*)), and *sxc^H537A^* homozygous adults were lysed, and the lysates were used for immunoblotting with anti-*O*-GlcNAc (RL2) or anti-OGT antibodies. The blots were normalized with either anti-actin or anti-α-tubulin antibodies, respectively. *C*, WT (*w^1118^*; *top*) or *sxc^H537A^* (*bottom*) homozygous embryos were immunostained with anti-*O*-GlcNAc antibody (RL2). Shown are stage 9–11 embryos of each of the genotypes.

**Figure 4.**
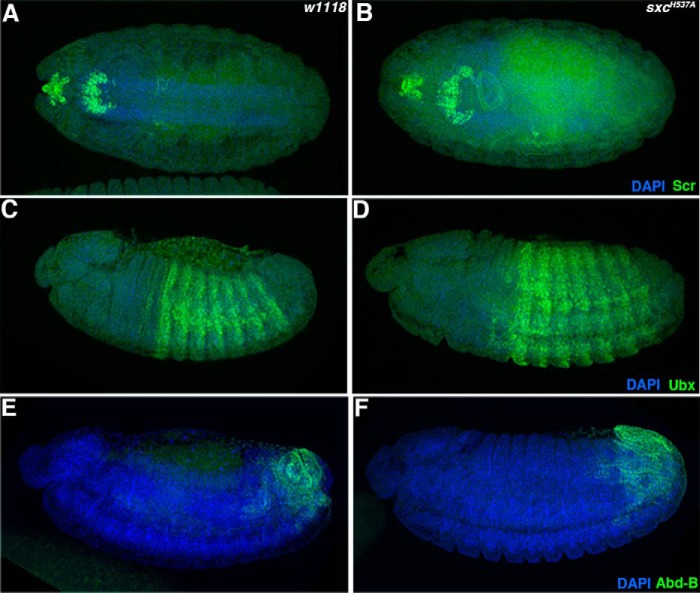
**Reduced O-GlcNAc levels in *sxc^H537A^* mutants does not affect *Hox* gene expression pattern.** Stage 13–14 WT (*w^1118^*; *A*, *C*, and *E*) or *sxc^H537A^* (*B*, *D*, and *F*) embryos were immunostained with anti-Scr (*A* and *B*), anti-Ubx (*C* and *D*), or anti-Abd-B (*E* and *F*) antibodies. The expression domains of all of these *Hox* genes tested remained unchanged. All of the embryos are aligned along the anterior-posterior axis with the anterior to the *left*. Embryos are depicted in either dorsal (*A* and *B*) or lateral (*C–F*) views.

We probed the levels of global *O*-GlcNAc and OGT in the *sxc^H537A^* mutant embryos (Fig. 3*A*) and adults (Fig. 3*B*). Immunoblots with a commercial *O*-GlcNAc antibody (RL2) revealed a large reduction in protein *O*-GlcNAcylation in F2 embryos that lack WT maternal and zygotic contribution and in adults (Fig. 3, *A* and *B*). However, OGT protein levels are comparable between WT and *sxc^H537A^* mutant embryos or adults (Fig. 3, *A* and *B*). Immunostaining *sxc^H537A^* homozygous embryos using RL2 antibody revealed a global reduction in *O*-GlcNAc levels as compared with the WT embryos (Fig. 3*C*). However, the reduced *O*-GlcNAc levels in *sxc^H537A^* embryos do not lead to a change in the expression domains of Hox proteins, Scr, Ubx, and Abd-B, as compared with the WT (Fig. 4).

**Figure 5.**
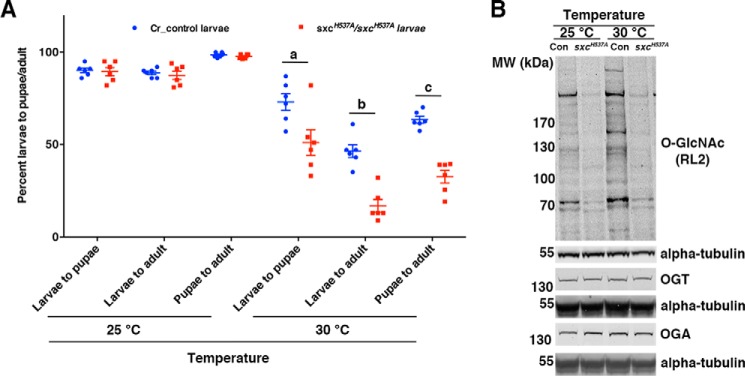
**Reduced *O*-GlcNAc levels in *sxc^H537A^* mutants leads to increased larval/pupal lethality at higher temperature.** *A*, lethality at higher temperature is increased in *sxc^H537A^* homozygotes. Either *Cr control* or *sxc^H537A^* F1 larvae (25 per vial, 100 larvae per experiment; *n* = 6) were transferred to fresh food vials at 25 or 30 °C, and the numbers of pupae formed and adults eclosed were counted. Development to pupae/adults from larvae or to adulthood from pupae was significantly reduced in *sxc^H537A^* homozygotes compared with *Cr control* flies (*a* and *b*, *p* < 0.001; *c*, *p* < 0.05; *t* test with Holm–Sidak correction). *B*, *O*-GlcNAc levels remain unaltered at higher temperature in *sxc^H537A^* embryos. Age-matched stage 16 *Cr control* or *sxc^H537A^* embryos were collected at either 25 or 30 °C, dechorionated, lysed, and subjected to SDS-PAGE and immunoblotted with anti-*O*-GlcNAc (RL2), anti-OGT, or anti-OGA antibodies. The blots were normalized with either rabbit anti-α-tubulin (*O*-GlcNAc blot), mouse anti-α-tubulin (OGT and OGA blots), or antibodies. This blot is representative of three biological replicates.

To assess whether reduced *O*-GlcNAc levels in the *sxc^H537A^* mutants resulted in defects during larval/pupal development, *Cr control* (generation outlined under “Experimental procedures”) or *sxc^H537A^* mutant L1 larvae were transferred onto fresh food vials, and the numbers of pupae formed as well as adults eclosed were evaluated. When the larvae were collected from embryos grown at 25 °C, there was no difference in the percentage of larvae developing to pupae or adults between *Cr control* and *sxc^H537A^* mutants (Fig. 5*A*). Given that increased temperature affects the viability of *sxc* null flies (46), pupae formation and adult eclosion was also assessed at 30 °C. Larval to pupal or adult development was significantly affected in *sxc^H537A^* mutants as compared with *Cr control* flies at 30 °C (Fig. 5*A*). Whereas 73 and 46% *Cr control* larvae develop into pupae and adults, respectively, only 51 and 17% of *sxc^H537A^* mutant larvae develop to pupae and adults (Fig. 5*A*). Pupal to adult development was 63 and 33% in *Cr control* and *sxc^H537A^* mutants, respectively (Fig. 5*A*). The increased lethality of *sxc^H537A^* homozygotes was associated with the inability to increase total *O*-GlcNAc levels at 30 °C as compared with the *Cr control* (Fig. 5*B*), which appears to be independent of OGT or OGA protein levels (Fig. 5*B*). In summary, it appears that the ability to increase *O*-GlcNAc levels with an increase in temperature during *Drosophila* development is protective to the organism. We next went on to investigate whether global reduction in *O*-GlcNAc levels in the *sxc^H537A^* affects dHcf function.

### Hypomorphic OGT phenotype is enhanced on reducing levels of transcriptional modulators

**Figure 6.**
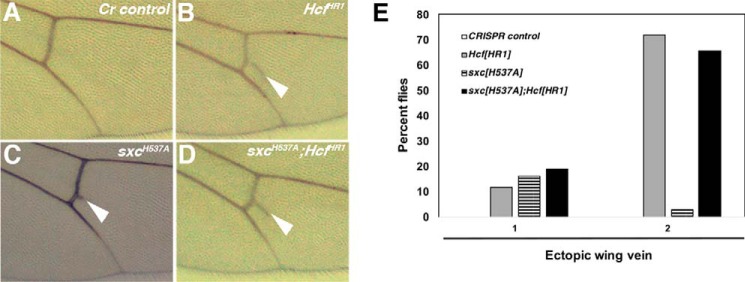
**Ectopic wing vein phenotype of *dHcf^HR1^* mutants is not enhanced in *sxc^H537A^* mutants.** *A*, image of the wing of an adult fly from the *Cr control* stock. There is no ectopic wing vein material seen arising from the posterior cross-vein in any of the control fly wings. Also marked are the longitudinal veins (L4 and L5). *B*, in *Hcf^HR1^* homozygotes, ectopic wing vein material is seen deposited in most flies, marked by the *white arrowhead. C*, in *sxc^H537A^* homozygotes, this phenotype is not as penetrant. *D*, the number of *sxc^H537A^; Hcf^HR1^* double homozygous flies having ectopic wing vein phenotype is comparable with penetrance seen in *Hcf^HR1^* homozygotes. *E*, the number of adult flies having ectopic wing vein deposition arising from the posterior cross-vein from *Cr control* (*white bar*), *Hcf^HR1^* homozygotes (*gray bar*), *sxc^H537A^* homozygotes (*hatched bar*), and *sxc^H537A^; Hcf^HR1^* double homozygotes (*black bar*) were counted. The graph represents the percentage of flies from each of the above genotypes having the ectopic wing vein in either one or both of the wings. None of the *Cr control* flies have ectopic wing veins, whereas quite a high percentage of *Hcf^HR1^* homozygotes display this phenotype. The proportion of *sxc^H537A^; Hcf^HR1^* double homozygotes have similar levels of the ectopic wing vein phenotype.

One of the striking phenotypes observed in 22% of *sxc^H537A^* adults was an ectopic wing vein emerging from the posterior cross-vein (Fig. 6, *A* and *B*). Homozygotes for *dHcf* null allele, *dHcf^HR1^*, display a similar phenotype (43). We therefore assessed the genetic interaction between the *dHcf^HR1^* null allele and the *sxc^H537A^* hypomorph, given that dHcf is a well-characterized *O*-GlcNAcylated protein in humans (41) and *Drosophila* (22). A previous report has characterized the genetic interaction between *skd^1^* (a hypomorphic recessive lethal *skd* allele) and the *dHcf^HR1^* allele resulting in enhancement of the ectopic wing vein phenotype, along with extra scutellar bristle and genitalia rotation phenotypes (43). There was no enhancement of the ectopic wing vein phenotype in *sxc^H537A^; dHcf^HR1^* double homozygotes (Fig. 6, *D* and *E*) compared with *dHcf^HR1^* homozygotes (Fig. 6, *C* and *E*). Moreover, the genitalia rotation phenotype was not observed in any of the genotypes tested.

**Figure 7.**
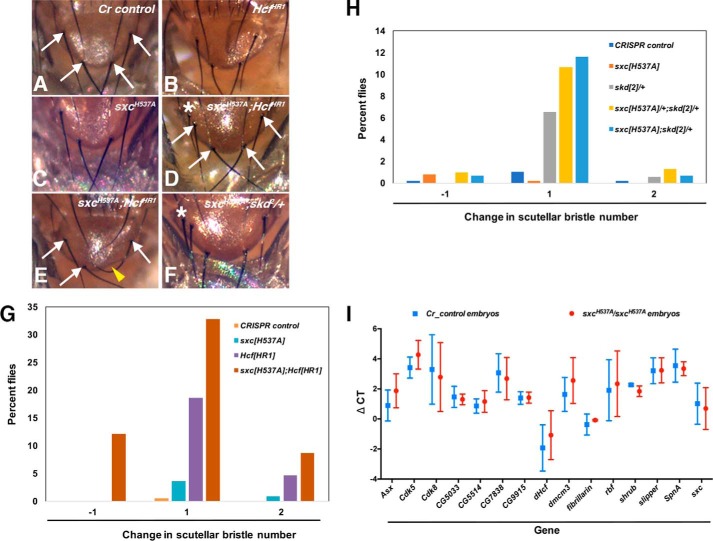
***sxc^H537A^* extra scutellar bristle phenotype is enhanced in *Hcf* null background.** *A*, *Cr control*; *B*, *Hcf^HR1^* homozygotes; *C*, *sxc^H537A^* homozygotes; *D*, *sxc^H537A^; Hcf^HR1^* double homozygotes; *E*, *sxc^H537A^; Hcf^HR1^* double homozygotes. *F*, *sxc^H537A^; skd^2^*/+ flies were treated with Flynap, and scutellar images were captured. The *white arrows* mark the four scutellar bristles. Homozygous *Hcf^HR1^* or *sxc^H537A^* homozygotes predominantly possess four scutellar bristles. However, in *sxc^H537A^; Hcf^HR1^* double homozygotes, over half of the flies have either extra (*D*) or missing (*E*) scutellar bristle(s). Flies of the genotype *sxc^H537A^; skd^2^/*+ also have slightly increased extra scutellar bristle phenotype (*F*). The extra scutellar bristle is marked with an *asterisk* in *D* and *F*. The *yellow arrowhead* marks the missing scutellar bristle in *E. G*, the number of scutellar bristles in adult flies from *Cr control* (*orange bars*, *n* = 199), *Hcf^HR1^* (*blue bars*, *n* = 43), *sxc^H537A^* (*purple bars*, *n* = 111), and *sxc^H537A^; Hcf^HR1^* double homozygotes (*brown bars*, *n* = 58) were counted. The graph represents the percentage of flies from each of the above genotypes having either one less (−*1*) or one (*1*) or two (*2*) more than the four scutellar bristles mostly observed in control flies. All of the control (*Cr control*) flies have only four scutellar bristles with minor deviation toward an extra one or two scutellar bristles in *Hcf^HR1^* or *sxc^H537A^* homozygotes. However, a significant proportion of *sxc^H537A^; Hcf^HR1^* double homozygotes have varying scutellar bristle numbers. *H*, the number of scutellar bristles in adult flies from *Cr control* (*dark blue*, *n* = 492), *sxc^H537A^* (*orange bars*, *n* = 507), *skd^2^*/+ (*gray bars*, *n* = 184), *sxc^H537A^*/+*; skd^2^*/+ (*yellow bars*, *n* = 309), and *sxc^H537A^; skd^2^*/+ (*light blue bars*, *n* = 146) flies were counted. The graph represents the percentage of flies from each of the above genotypes having either one less (−*1*) or one (*1*) or two (*2*) more than the four scutellar bristles mostly observed in control flies. There is a modest increase in the percentage of *sxc^H537A^*/+*; skd^2^*/+ or *sxc^H537A^; skd^2^*/+ flies having extra scutellar bristles as compared with *skd^2^*/+ flies. *I*, quantitative real-time PCR was performed to detect the transcripts potentially downstream of dHcf apart from *sxc* and *dHcf* transcripts. The graph represents the Δ*C*_T_ values of the respective transcripts in either *Cr control* (*blue squares*) or *sxc^H537A^* (*red circles*) stage 7–11 embryos. The experiments were repeated three times, and no significant difference was observed in the levels of any of the transcripts assessed (*t* test with Holm–Sidak correction).

There are four scutellar bristles in most *Drosophila* species (49). In a previous study, *skd^1^* heterozygotes were found to have normal bristle numbers, whereas about a third of *skd^1^* heterozygotes in a *dHcf^HR1^* background possessed extra scutellar bristles (43). In our experiments, all of the *Cr control* flies had the normal component of four scutellar bristles (Fig. 7, *A*, *G*, and *H*). On examining *sxc^H537A^* homozygotes (*n* = 111), about 5% of the flies were found to have either one or two extra scutellar bristles (Fig. 7, *C* and *G*). In *dHcf^HR1^* homozygotes, the percentage of flies with extra scutellar bristles was 18% (Fig. 7, *B* and *G*). Interestingly, 41% of *sxc^H537A^; dHcf^HR1^* double homozygotes (*n* = 58) had one or two extra scutellar bristles, whereas 12% were missing a scutellar bristle (Fig. 7, *D*, *E*, and *G*). The defect in flies scored for a missing bristle was the complete loss of the mechanosensory organ as opposed to accidental bristle damage (Fig. 7*E*). These data therefore demonstrate an interaction between the *sxc^H537A^* and *dHcf^HR1^* alleles, specifically in the determination and/or function of the sensory organ precursor (SOP) cells essential for bristle formation. Furthermore, we also investigated whether the deregulation of scutellar bristle number is affected by *PcG* (Polycomb; *Pc*) and *TrxG* (brahma; *brm*) genes (Table 2). On reducing one copy of *Pc* (*Pc^1^*, an amorphic recessive lethal allele) in either *sxc^H537A^*/+ or *sxc^H537A^* background, a normal number of scutellar bristles was observed, indicating no genetic interaction with respect to this phenotype (Table 2). However, the super sex combs phenotype (sex combs in the second and third pairs of thoracic legs) observed in *Pc^1^*/+ flies (21% of all males scored) was enhanced in an *sxc^H537A^*/+ (56%) or *sxc^H537A^* (66%) background, revealing a role of the catalytic activity of *sxc* in Polycomb function (Table 3). *Cr control* or *sxc^H537A^* flies did not exhibit the super sex combs phenotype (Table 3). On performing a genetic interaction between *sxc^H537A^* and *brm^2^* alleles, only a small percentage of *sxc^H537A^*/+*; brm^2^*/+ (5%) or *sxc^H537A^; brm^2^*/+ (4.8%) flies were found to have the scutellar bristle phenotype (Table 2).

**Table 2:** **Scutellar bristle phenotype of *sxc^H537A^* mutants is not affected by reduction of Polycomb function** Flies of the respective genotypes were scored for the number of scutellar bristles. The percentage of flies exhibiting either less or more than the normal scutellar bristle number of four are listed.

Genotype	Number of flies scored	Percentage of flies with decreased scutellar bristle number	Percentage of flies with increased scutellar bristle number
*Cr control*	388	0.3	1
*sxc^H537A^*	302	1	0.3
*Pc^1^*/+	247	0.4	0
*sxc^H537A^*/+*; Pc^1^*/+	197	0	0.5
*sxc^H537A^; Pc^1^*/+	150	2	0
*brm^2^*/+	99	0	0
*sxc^H537A^*/+*; brm^2^*/+	302	0.3	5
*sxc^H537A^; brm^2^*/+	104	0	4.8

**Table 3:** **Super sex combs phenotype of *Pc^1^* is enhanced in *sxc^H537A^* background** Males of the respective genotypes were scored for the presence of sex combs on second and third thoracic legs. Percentage of flies exhibiting the super sex combs phenotype are listed.

Genotype	Number of males scored	Percentage of males, super sex combs phenotype
*Cr control*	203	0
*sxc^H537A^*	153	0
*Pc^1^*/+	124	21
*sxc^H537A^*/+*; Pc^1^*/+	115	56
*sxc^H537A^; Pc^1^*/+	80	66

To investigate whether reduced *O*-GlcNAc levels in the *sxc^H537A^* homozygotes also impinge upon *skd* function or vice versa, interaction between *sxc^H537A^* and a hypomorphic recessive lethal *skd* allele, *skd^2^* (the *skd^1^* stock is not publicly available), was assessed. About 7% of the *skd^2^* heterozygotes (*n* = 184) displayed extra scutellar bristles. Slightly higher abnormal scutellar bristle numbers were observed in both *sxc^H537A^/*+; *skd^2^/*+ double heterozygotes (13%, *n* = 309; Fig. 7*H*) and *sxc^H537A^; skd^2^*/+ flies (13%, *n* = 146; Fig. 7, *F* and *H*), indicating a genetic interaction between the *sxc^H537A^* and *skd^2^* alleles, albeit to a lesser extent than that observed between the *sxc^H537A^* and *dHcf^HR1^* alleles. Adults of the genotype *sxc^H537A^; skd^2^*/+*; dHcf^HR1^* could not be derived, implying that loss of OGT and dHcf activity in *skd* heterozygotes leads to developmental lethality.

In light of the genetic interaction between *sxc^H537A^* and *dHcf^HR1^* alleles, we investigated whether dHcf function is affected in *sxc^H537A^* mutants. Knockdown of *dHcf* in S2 cells was previously reported to lead to transcriptional up-regulation of *fibrillarin* and *CG5033* (50). There is also evidence that dHcf interacts with *Drosophila* elongation factors dE2F1 and dE2F2 (51). Data from human cell lines implicate a role for HCF1 in transcriptional control of E2F-bound genes (52). Transcription of several genes (Table S4), including *ASXL*, *CDK5*, and *CDK8*, is deregulated on HCF1 knockdown (52). We investigated the changes in transcript levels of dHcf/HCF1 downstream targets derived from both of these studies (50, 52) in *Cr control* and *sxc^H537A^* embryos. The transcript levels of all of the dHcf/HCF1 downstream targets investigated remained unchanged when compared with those in *Cr control* embryos (Fig. 7*I*). In summary, these data implicate *sxc*, *dHcf*, and *skd* in a common pathway that is responsible for scutellar bristle determination. Nevertheless, the molecular details of how reduced *O*-GlcNAc levels in the *sxc^H537A^* mutants contribute to this phenotype remain to be investigated.

## Discussion

Using CRISPR/Cas9 technology, we have been able to produce an important tool in the form of a hypomorphic *sxc* mutant. This is particularly useful, given that *sxc* is a maternal effect gene and that its genomic locus impedes production of mutants that lack maternal as well as zygotic gene products using the Flipase/FRT system (53). Previous studies have circumvented this hurdle using various transgenic approaches (8, 23, 46). However, nonendogenous, constitutive expression of transgenic OGT can lead to artifacts. In addition, our previous observation that minimal OGT glycosyltransferase activity is sufficient to sustain *Drosophila* development through multiple generations was an added impetus to produce catalytically deficient OGT mutants in an otherwise endogenous background (23). The *sxc^H537A^* mutant provides a platform to investigate the role of OGT catalytic activity in *Drosophila* development. Utilizing a restriction assay to screen for potential mutants, we have harnessed the CRISPR/Cas9 gene-editing technology to create precise *sxc* point mutations. We were able to produce two precise *sxc* point mutations, *sxc^H537A^* and *sxc^K872M^*, at an efficiency of 25%, starting from fertile injected males for each of the mutations.

Phenotypic analysis of the *sxc^K872M^* mutant that codes for a catalytically dead mutant could not be pursued because this mutation is recessive lethal. This observation is supported by the fact that the previously published *sxc^1^* or *sxc^6^* mutant alleles cannot be complemented by the *sxc^K872M^* allele. In addition, we were able to derive null alleles from the H537A gRNA injections that also did not complement *sxc^1^* or *sxc^6^* lethality. These results establish the specificity of the gRNAs used in our CRISPR/Cas9 approach to create the *sxc* point mutations. Specificity of the mutagenesis was also highlighted by the significant reduction of *O*-GlcNAc levels in *sxc^H537A^* homozygotes. The lack of derepression of *Hox* genes in *sxc^H537A^* F2 embryos reiterates our earlier finding that a minimal level of *O*-GlcNAcylation is sufficient to support *Drosophila* development (23). The data obtained in the current work eliminate the potential artifacts of overexpression and the possibility that WT and mutant forms of OGT form heteromeric complexes. In this scenario of significantly reduced global *O*-GlcNAc that does not lead to *Hox* gene derepression, it will be interesting to investigate the dynamics of Ph *O*-GlcNAcylation and consequently its aggregation/loss of function (8). This is relevant because the loss of Ph function leads to derepression of *Hox* genes in embryos and larval imaginal discs (16, 54).

The reduced levels of protein *O*-GlcNAcylation in *sxc^H537A^* homozygotes are associated with larval and pupal lethality at elevated temperatures. It has previously been reported that elevated temperature leads to lethality during embryogenesis in maternal or zygotic *sxc* mutants (46). The endogenous *sxc^H537A^* mutant has enabled us to identify the specific requirement of *catalytic activity* of OGT as opposed to the OGT *interactome*, at post-embryonic stages of development. It opens up the possibility that the *O*-GlcNAc modification, akin to glycosylation in the secretory pathway, is essential for stabilizing misfolded proteins at higher temperatures. Heat stress in mammalian cells is associated with increased cellular *O*-GlcNAc levels. Reducing OGT catalytic activity by genetic or chemical means renders the cells more susceptible to thermal stress (55, 56). Heat-stressed *OGT*^−/−^ mouse embryonic fibroblasts have reduced levels of specific heat-shock proteins (57). Downstream of OGT/*O*-GlcNAc cycling, the levels of these heat-shock proteins are proposed to be regulated by GSK3β-dependent phosphorylation of heat-shock factor 1 (57). Several proteins with diverse functions were demonstrated to be hyper-*O*-GlcNAc–modified and up-regulated on heat stress in monkey fibroblasts (58). Heat stress–induced heat-shock protein 70 has been described to bind to *O*-GlcNAcylated proteins, preventing their misfolding (59). Increased hsp70 levels on heat stress are probably downstream of *O*-GlcNAcylated Sp1 (60). Nevertheless, the mechanistic details of how *O*-GlcNAc–dependent thermoprotection occurs in *Drosophila* require further analyses.

Scutellar bristles arise from progenitors in the larval wing imaginal disc epithelium known as SOPs. Clusters of cells that express proneural genes of the *achaete-scute* (*ac-sc*) complex are subjected to selection by Notch-Delta signaling–mediated lateral inhibition. This process leads to specification of SOPs (61–64). Once specified, the SOPs go on to differentiate into mechanosensory organs via a complex, orchestrated pathway (65). A GATA-1 family transcription factor, Pannier (*Pnr*), is an activator of *ac-sc*, specifically required for the specification of the dorsocentral bristles that are nonscutellar mechanosensory organs (66). The extra bristle phenotype of the *pnr^D1^* allele is enhanced by the *Pc^1^* allele, implying PcG-mediated control of SOP determination (66). However, we do not observe an interaction between *Pc^1^* and *sxc^H537A^* with respect to bristle numbers in the scutellum. Moreover, the phenotype observed in *sxc^H537A^; Hcf^HR1^* double homozygotes is one wherein there is increased variation in the number of scutellar bristles, with some flies also having a reduced number of bristles. These observations therefore imply that the specification of scutellar SOPs in *sxc^H537A^* flies is not via the influence of OGT catalytic activity on PcG function.

The extra scutellar bristle phenotype is enhanced significantly in *sxc^H537A^; Hcf^HR1^* double homozygotes when compared with either *sxc^H537A^* or *Hcf^HR1^* homozygotes. This phenotype is also enhanced in *Hcf^HR1^* homozygotes that have a single copy of the *skd^1^* allele (43). However, we observe a weaker genetic interaction between *sxc^H537A^* and *skd^2^* alleles as compared with the interaction between *sxc^H537A^* and *Hcf^HR1^*. This implies that the pathways potentially affected by reduced *O*-GlcNAc levels in *sxc^H537A^* flies are able to tolerate the presence of a hypomorphic copy of *skd* more effectively than an *Hcf^HR1^* null background. None of the other phenotypes described for the *skd^1^*/+*; Hcf^HR1^* flies were recapitulated in either *sxc^H537A^; Hcf^HR1^* or *sxc^H537A^; skd^1^*/+ animals, indicating specific roles for *O*-GlcNAc in dHcf and/or Mediator complex function. Nevertheless, reduction in *O*-GlcNAc levels is not tolerated in animals both having reduced skd levels and lacking dHcf. Interestingly, point mutations in human *OGT* and *MED12*, another Mediator component, co-segregate in individuals affected with X-linked intellectual disability (XLID) (13, 14). Mutations have also been identified in human *HCF1* that are associated with X-linked mental retardation (67, 68). Moreover, rare variants of both *MED12* and *HCF1* were shared only by the affected siblings in a family affected by a severe form of XLID (69). We observe a common pathway being affected when orthologs of XLID genes are used in genetic interaction experiments. Therefore, the scutellar bristle number phenotype is potentially a readout in *Drosophila* to genetically dissect the contribution of OGT/*O*-GlcNAc function in XLID.

In conclusion, we have demonstrated successful generation of catalytically hypomorphic *sxc* mutants using a simple, transferable assay to screen for mutagenesis by CRISPR/Cas9 gene editing. Analysis of the *sxc^H537A^* thus obtained has helped uncover several phenotypes that are a result of a reduction in protein *O*-GlcNAcylation. Either the reduced *O*-GlcNAcylation of dHcf or conversely decreased dHcf function impinging upon OGT function(s) affects normal scutellar bristle numbers. Moreover, *Drosophila* embryos possess a dynamic *O*-GlcNAcome that could contribute to phenotypes described in this study and others that remain to be discovered (22, 70, 71). Apart from other applications, the hypomorphic *sxc^H537A^* mutant is a tool that can be used to investigate the role of dHcf *O*-GlcNAc, potentially developed as a model to investigate the role of OGT in XLID and investigate *O*-GlcNAc occupancy in the Ph Ser/Thr-rich stretch. Moreover, investigating the *O*-GlcNAcome in *sxc^H537A^* mutants would help in narrowing down key transducers of *O*-GlcNAc signaling in *Drosophila* development. This analysis will be particularly informative in eliminating the functionally inconsequential *O*-GlcNAcylation events and establish the role of *O*-GlcNAc signaling in *Drosophila* development.

## Experimental procedures

### Drosophila genetics, scutellar imaging, and immunostaining

The following stocks from the Bloomington Drosophila Stock Centre were used: *w^1118^* WT, *vasa::Cas9* (BL51323), *Hcf^HR1^*, *skd^2^/TM6*, *brm^2^/TM6*, and *Pc^1^/TM1*. CRISPR/Cas9 injections were performed at the University of Cambridge fly facility into embryos from the *vasa::Cas9* line (Bloomington stock: 51323). Microinjections were carried out with a mixture of 100 ng/μl gRNA plasmid with 300 ng/μl repair construct mix. Injected founder male flies were crossed with *IF/CyO; MKRS/TM6* balancer stock. At least 10 male F1 sxc*/*CyO* potential germ line mutants were crossed again with *IF/CyO; MKRS/TM6* virgins. This ensured the outcrossing of the *vasa::Cas9*-carrying X chromosome. The F1 males were then snap-frozen for genotyping as outlined below. Stocks of either *sxc^H537A^/CyO* or *sxc^K872M^/CyO* were established from F2 progeny of sequence-confirmed mutants. Furthermore, the genotype of *sxc^H537A^* homozygotes derived from the *sxc^H537A^/CyO* stock was confirmed. In addition, all of the predicted off-target sites were PCR-amplified and checked for the presence of any lesions compared with the genomic DNA from the BL51323 line. None of the predicted off-target sites were found to have mutations. To perform Western blots with whole flies, either WT or the *sxc^H537A^* flies were snap-frozen and processed as outlined below. The control flies (*Cr control*) were derived by crossing the flies from the stock used for microinjection (Bloomington Stock: BL51323) using a similar crossing scheme as that used to derive the *sxc^H537A^* homozygotes. This ensured maintenance of the genetic background and the loss of the *vasa::Cas9*-carrying X chromosome.

The number of scutellar bristles was assessed in the genotypes *Cr control*, *sxc^H537A^*, *Hcf^HR1^*, *skd^2^*/+, *sxc^H537A^; Hcf^HR1^*, *sxc^H537A^; skd^2^*/+, *sxc^H537A^*/+*; skd^2^*/+, *brm^2^*/+, *sxc^H537A^*/+*; brm^2^*/+, *sxc^H537A^; brm^2^*/+, *Pc^1^*/+, *sxc^H537A^*/+; *Pc^1^*/+, and *sxc^H537A^; Pc^1^*/+, using a Motic SMZ microscope. Images from representative flies treated with FlyNap (Carolina Biological Sciences) were acquired using a Leica E24 HD dissection microscope. The presence of sex combs on second and third thoracic legs was scored for the genotypes *Cr control*, *sxc^H537A^*, *Pc^1^*/+, *sxc^H537A^*/+*; Pc^1^*/+, and *sxc^H537A^; Pc^1^*/+, using a Motic SMZ microscope.

Fixing and immunostaining of embryos was performed as described previously (72). The following antibodies were used: mouse anti-*O*-GlcNAc (1:250, RL2, Abcam) and mouse antibodies from the Developmental Studies Hybridoma Bank (anti-Scr (1:50), anti-Abd-B (1:50), and anti-Ubx (1:50)) with the respective fluorescent secondary antibodies (Invitrogen). Microscopic images were obtained with Leica SP8 confocal microscope and processed using Volocity (Improvision) software.

### Cloning and restriction fragment length polymorphism assay to detect mutants

gRNA sites were chosen using the website crispr.mit.edu,^4^ and annealing oligonucleotides were designed with the appropriate overhangs and cloned into the BpiI-cut pCFD3-dU63gRNA vector (Table S1). Inserts were confirmed by DNA sequencing.

Repair templates were generated by PCR of either a 2160-bp (H537A) or a 2063-bp (K872M) region of the *Drosophila* genome from S2 cell genomic DNA using GoTaq G2 polymerase (Promega). The PCR product was cloned into pGEX6P1 plasmid. The insert sequence was confirmed by DNA sequencing. The desired mutation, in addition to the silent mutations, was introduced by site-directed mutagenesis following the Stratagene QuikChange mutagenesis kit but using KOD Hot Start polymerase (Novagen) and subsequently confirmed by DNA sequencing.

To assess and confirm generation of CRISPR/Cas9 gene editing, candidate homo-/heterozygous flies were frozen in Eppendorf tubes and homogenized in 50 μl of squishing buffer (10 mm Tris-HCl, pH 8, 1 mm EDTA, 25 mm NaCl, and 200 μg/ml freshly added Proteinase K (Roche Applied Science)). The homogenate was incubated at 37 °C for 30 min, followed by inactivation of Proteinase K at 95 °C for 3 min, and centrifuged. 1 μl of supernatant was used per 25-μl PCR. 5 μl of PCRs was digested with TaqI (H537A PCR) or XhoI (K872M PCR), followed by electrophoresis of the digested products. Samples that were resistant to TaqI and XhoI digestion were sequenced. A second PCR was performed on potential heterozygous CRISPR mutants with primer pairs out with the repair construct to confirm that the observed sequencing result was not due to random integration of the repair plasmid. The second PCR product was also sequenced. To determine any potential mutagenesis at any of the predicted off-target sites, PCRs were performed with the requisite primers (Table S2), followed by sequencing.

### Eclosion rate experiments

For eclosion rate experiments, *Cr control* or *sxc^H537A^* homozygote flies were transferred to apple juice agar plates thinly smeared with yeast paste at either 25 or 30 °C. After an overnight collection, 25 F1 larvae were transferred to fresh food vials. Four such vials were set up per biological replicate (*n* = 6; a total of 600 F1 larvae were thus scored for each genotype). The number of pupae formed was assessed by counting the number of pupal cases per food vial. In addition, the number of adult flies eclosing from each vial was also recorded. We report the percentage of F1 larvae forming pupae/adults and the number of pupae giving rise to adults. *t* tests were performed for statistical analyses.

To harvest embryos for Western blotting, embryos were collected for 1 h and further aged (to stage 16) for either 13.5 or 11 h at 25 or 30 °C, respectively, before dechorionating and snap-freezing the embryos. The frozen embryos were subjected to Western blot analysis as outlined below.

### Western blotting

To prepare total embryo lysates, embryos were collected on apple juice agar plates at 25 °C overnight (0–16 h). The embryos thus collected were dechorionated with bleach and snap-frozen in dry ice. The frozen embryos were homogenized in lysis buffer (50 mm Tris-HCl, pH 8.0, 150 mm NaCl, 1% Triton X-100, 1 μm GlcNAcstatin C, 5 mm sodium fluoride, 2 mm sodium orthovanadate, 1 mm benzamidine, 0.2 mm phenylmethylsulfonyl fluoride, 5 μm leupeptin, and 1 mm DTT). For Western blots, five anesthetized adult flies were frozen on dry ice. The frozen flies were homogenized in 50 μl of lysis buffer, followed by the addition of an equal volume of 3× SDS Laemmli buffer. Lysates were then heated for 5 min at 95 °C and centrifuged at 16,000 × *g* for 10 min, and supernatants were collected. Protein concentrations were estimated using the 660-nm protein assay (Thermo Scientific). 30 μg of the crude lysate was subjected to SDS-PAGE and transferred onto nitrocellulose membrane before immunoblotting with RL2 (1:1000; Abcam), rabbit anti-OGT (H-300, 1:1000; Santa Cruz Biotechnology, Inc.), rabbit anti-OGA (1:1000; Sigma), mouse anti-α-tubulin (1:10,000; DSHB), and/or rabbit anti-actin (1:5000; Sigma) and the respective IR dye conjugated secondary antibodies (LI-COR or Life Technologies; 1:10,000).

### Quantitative real-time PCR

Quantitative real-time PCR was performed with *Cr control* and *sxc^H537A^* homozygous embryos. *Cr control* and *sxc^H537A^* were transferred to apple juice agar plates thinly smeared with yeast paste at 25 °C. Fresh plates were used to collect embryos for 2 h. The plates were then changed, and the embryos were allowed to age for 3 h. RNA isolation (Qiagen RNAeasy Plus kit), quantification (Nanodrop), and cDNA generation (Bio-Rad Iscript cDNA synthesis kit) were then performed as per the manufacturer's instructions. cDNA equivalent to 100 pg of input total RNA was subjected to quantitative real-time PCR (Quanta Biosciences) in a Bio-Rad CFX Connect system. Primers used for dHcf downstream targets (Table S4) were from either published literature (50) or an online tool for *Drosophila* primers (73). The reported threshold cycle (*C*_T_) values were used to compute Δ*C*_T_ values as described (74). Three biological replicates were used to determine the Δ*C*_T_ values, and *t* tests with the Holm–Sidak method to correct for multiple comparisons were used for statistical analysis.

## Author contributions

D. M., A. T. F., and D. M. F. v. A. conceived the study. D. M. performed the *Drosophila* experiments and phenotypic analyses. A. T. F. performed molecular biology. D. M., A. T. F., and D. M. F. v. A. interpreted the data and wrote the manuscript.

## Footnotes

4 Please note that the JBC is not responsible for the long-term archiving and maintenance of this site or any other third party hosted site.

## Footnotes

3 The abbreviations used are:

OGT: *O*-GlcNAc transferase
OGA: *O*-GlcNAcase
DSB: dsDNA break
gRNA: guide RNA
sgRNA: single guide RNA
bp: base pair(s)
kb: kilobase pair(s)
SOP: sensory organ precursor
XLID: X-linked intellectual disability.
